# Trends in inequality in length of life in India: a decomposition analysis by age and causes of death

**DOI:** 10.1186/s41118-017-0022-6

**Published:** 2017-07-10

**Authors:** Abhishek Singh, Ankita Shukla, Faujdar Ram, Kaushalendra Kumar

**Affiliations:** 1 Department of Public Health & Mortality Studies, International Institute for Population Sciences, Mumbai, 400 088 India; 2 International Institute for Population Sciences, Mumbai, 400 088 India

**Keywords:** Inequality in length of life, Decomposition by age and causes of death, Gini coefficient, Sample Registration System, Global Burden of Disease Study, India

## Abstract

**Background:**

Studies dealing with trends in inequality in length of life in India are rare. Studies documenting the contribution of age and causes of death to the inequality in length of life are more limited.

**Objective:**

The study aims to examine the trends in inequality in length of life in India and 15 major states of India and to decompose the inequality in length of life into the contributions of age and causes of death.

**Method:**

We use life table Gini coefficient (*G*_0_) to measure the inequality in length of life. We use the formulae developed by Shkolnikov, Andreev, and Begun (DR 8(11):305–358, 2003) to decompose the differences between Gini coefficients by age and cause of death.

**Result:**

The *G*_0_ for men has declined from 0.32 in 1981 to 0.19 in 2011. For women, *G*_0_ has decreased from 0.31 in 1981 to 0.22 in 2011. Mortality decline in the age group 0–1 year has contributed most to the decrease in *G*_0_. In contrast, mortality decline in 60+ has tended to increase the *G*_0_. The state-wide variations in the age-specific contributions to decrease in *G*_0_ were stark. The contribution of noncommunicable diseases to the male-female gap in *G*_0_ has increased between 1990 and 2010. Injuries at ages from 20 to 39 years also contributed to the male-female difference in *G*_0_ in 2010.

**Conclusion:**

Future studies must analyze inequality in life expectancy for assessing the performance of societies regarding length of life.

**Contribution:**

This is the first study that provides compelling evidence on inequality in length of life in India and its major states.

## Introduction

Life expectancy at birth (both sexes combined) in India has risen from 54 years in 1981 to 67 years in 2011(RGI 2013). Although the life expectancy has increased, the increase is not uniform across the different states and subpopulations of India. For example, life expectancy for males rose from 54 years in 1981 to 66 years in 2011. For females, it increased from 54 years to 69 years during the same period. Different states of India depict significant variations in trends in life expectancy at birth, with differences often years or more between individual states. Such trends in life expectancy at birth reveal widespread inequality in length of life.

Previous research also suggests that apart from socioeconomic, regional, and biological factors, there are some intangible factors which affect individual’s life expectancy irrespective of the group to which the individual belongs (Edwards and Tuljapurkar 2005; Wolfson and Rowe 2001). Even if all conditions are constant, not everybody dies at the same age, and consequently there may be inequality in length of life among individuals within subgroups. Hence, the level of life expectancy alone cannot provide the complete picture of mortality situation in a population. For all these reasons, it is important to examine the inequality in length of life systematically. Moreover, it is also important to understand the changes in inequality in length of life.

Systematic evidence on trends in inequality in length of life mostly comes from the high-income countries. Wilmoth and Horiuchi (1999) found that the Inter Quartile Range (IQR) fell dramatically in the U.S., Sweden, and Japan during the epidemiological transition after 1870, and flattened after 1950. However, the trends in life expectancy and inequality in life expectancy did not show the same patterns. Another study based on high-income countries showed that the variance in adult length of life fell rapidly during the epidemiological transition but remained stagnant afterward, with large differences across countries (Edwards and Tuljapurkar 2005). Smits and Monden (2009) in their study of high-income countries find large differences across countries in the level of within-country inequality in adult length of life. Shkolnikov et al. (2003) have also estimated the inequality in length of life for 32 countries in 1996–1999 and the associated changes in Gini coefficient in Japan, Russia, Spain, USA, and UK in 1950–1999.

The existing studies on life expectancy in India mostly discuss the differences in life expectancy across groups, for example, by sex or gender, rural-urban residence, and between states (James and Syamala 2010; Saikia et al. 2011; Sauvaget et al. 2011). A few studies have also attempted to decompose the changes in life expectancy into the contribution of mortality change in different age groups. The decomposition results reveal that recent improvements in life expectancy in India are mainly due to steeper mortality changes at the younger ages (Saikia et al. 2011; Singh and Ram 2003; Singh and Ladusingh 2010). Studies examining inequality in length of life in India are limited. Furthermore, there is no evidence on trends in inequality in length of life in India. We could come across only one study that has examined trends in temporary life expectancy between ages 0 and 60 in the Indian states (Saikia et al. 2011). The study by Saikia et al. (2011) revealed large reductions in inter-state inequality in temporary life expectancy between the periods 1970–1975 and 2000–2004. After significant gains in the life expectancy at birth during the 1970s and the 1980s in India, the increase has come to a worrying halt. Interestingly, the study by Saikia et al. (2011) did not examine the trends in inequality in different states and subpopulations of India. Furthermore, to the best of our knowledge, there is no study in India that has examined the individual-based measurement of inequality in length of life. Also, we could not come across any study that has decomposed the changes in inequality in length of life into the contribution of causes of deaths. Moreover, India comprises of 29 States and 7 Union Territories (UTs). The various States and UTs are at different levels of socioeconomic development. For example, the southern states—like Andhra Pradesh, Telangana, Karnataka, Kerala, and Tamil Nadu—are way ahead of other States in socioeconomic development. Also, various States/UTs are at different levels of demographic and epidemiological transitions. Therefore, an analysis of India and its states is likely to provide interesting patterns in inequality in length of life. Having said that, the present study examines the trends in inequality in length of life. Moreover, the present study also decomposes the changes in inequality in length of life into the contribution of mortality change in different age groups. Finally, we decompose the changes in inequality in length of life into the contribution of causes of death. We use time-series data from the India’s Sample Registration System and the Global Burden of Disease Study 1990 and 2010 to fulfil the objectives of the paper.

## Data and methods

We use data from the Sample Registration System (SRS) for the period 1981–2011 to examine trends in inequality in length of life in India and 15 major states of India. Sample Registration System (SRS) under the aegis of the Office of the Registrar-General of India (RGI) is the primary and continuous source of data on mortality and life tables for India and 16 major states. These states are Himachal Pradesh, Punjab, Haryana and Rajasthan (all from the north), Uttar Pradesh and Madhya Pradesh (from the central), Assam (from the Northeast), West Bengal, Odisha and Bihar (from the east), Gujarat and Maharashtra (from the west), and Andhra Pradesh, Karnataka, Kerala and Tamil Nadu (from the south). One of these 16 states, Himachal Pradesh, could not be included in the analysis as the data is deficient for Himachal Pradesh for the years 1980, 1981, and 1990. Hence, we restrict our analysis to India and 15 major states of India.

In SRS, the enumerators match the recorded vital events (as and when they occur) in a representative sample of rural and urban units with those from a bi-annual retrospective survey by independent supervisors (RGI 2013). The SRS provides information on age-specific death rates in different age groups up to 70+ starting from the year 1970. The SRS in the year 1995 disaggregated the age group 70+ into 70–74, 75–79, 80–84, and 85+ year age groups. Along with this, RGI also provides abridged life tables based on SRS data for 5-year periods starting from 1970 to 1975. Since the SRS-based life tables in the late 1990s and early 2000s are marked by inconsistencies in the age groups 1–4 (Saikia et al. 2010), we do not borrow the life expectancies from those life tables for our analysis. Instead, we use age-specific death rates published by the SRS to generate new life tables for our analysis.

For decomposing the inequality in length of life by age and causes of death, we use data from the two rounds of Global Burden of Disease (GBD) Study conducted in the years 1990 and 2010 (GBD 2013). GBD data is collected and analyzed by a consortium of more than 1000 researchers in over 100 countries. The GBD data captures premature death and disability from more than 300 diseases and injuries in 188 countries, by age and sex, from 1990 to the present, allowing comparisons over time, across age groups, and among populations (GBD 2013). Following the broad classification of the GBD study, we categorize the causes of deaths into three major categories: communicable, maternal, neonatal and nutritional conditions; noncommunicable diseases; injuries (GBD 2013).

### Measuring inequality in life expectancy

A wide array of statistics has been used by different researchers to measure inequality: interquartile range (IQR), variance (VAR), standard deviation (STD), the Theil entropy index (T), Gini coefficient, etc. Among them, Gini coefficient is considered as the most useful measure of inequality in length of life. As a formal construct, Gini coefficient has significant similarities with life expectancy, which allow applying similar methods for its calculation or decomposition (Shkolnikov et al. 2003).

Gini coefficient represents cumulative income share as a function of cumulative population share. Applying this device to mortality-by-age schedules, one can imagine a person’s years lived from birth to death to be “income” and cumulative death numbers to be “population”(Shkolnikov et al. 2003). The coefficient varies from “0” to “1”. It is equal to “0” if all people die at the same age and “1” if all individuals die at age “0” and one individual dies at an infinitely old age. Greater or lower values of Gini coefficient show a greater or lower magnitude of interindividual differences in length of life.

According to Hanada (1983), Gini can be defined as:

$$ {\mathrm{G}}_0=1-\frac{1}{e_0}.\kern0.5em {\displaystyle {\int}_0^{\infty }{\left[ l(x)\right]}^2\kern0.1em {d}_x} $$

A compact expression for the life table Gini by Hanada (1983) in its discrete form (Shkolnikov et al. 2003) is especially useful for practical calculations.

1 $$ {G}_x = 1-\kern0.5em \frac{1}{e_x{l}_x}{\displaystyle {\sum}_{t= x}^{w-1}\left[{\left({l}_{t+1}\right)}^2+\kern0.5em  A` x\Big({\left({l}_t\right)}^2-\kern0.5em {\left({l}_{t+1}\right)}^2\right]} $$

2 $$ \mathrm{where}\  A{`}_x=\kern0.5em \frac{\left[1-\left(2/3\right){q}_x+{C}_x\kern0.5em \left(2-{q}_x-\left(6/5\right)\kern0.5em {C}_x\right)\right]}{\left[2-{q}_x\right]} $$

$$ {C}_x=\kern0.5em {A}_x-1/2 $$

$$ {A}_x=\kern0.5em \frac{\left(\frac{L_x}{1}\right)-{l}_{x+1}}{l_x-{l}_{x+1}} $$

Formula (2) would not work in a proper way for *x* = 0 because during the first year of life *l*(*x*) falls much steeper than it can be predicted by a quadratic polynomial. The use of the formula by Bourgeois-Pichat (1951) solves the problem for age 0 and results in

$$ A{`}_0 = {A}_0\ \left[\ 1 - {q}_0\frac{3+0.831{A}_0}{2+{q}_0}\kern0.5em \right] $$

Estimating *A`*_*x*_ for the open age intervals using (2) is not possible. Shkolnikov et al. (2003) used 334 complete life tables for Japan, France, Sweden, and USA to estimate *A`*_85+_. They estimated *A`*_85+_ because in almost all the countries the last age group for mortality data is 85+. The 334 life tables for the aforementioned countries were obtained from the Berkeley Mortality Database (The Berkeley Mortality Database 2001). They used single-year mortality data starting from 85 to 110 years for estimating *A`*_85+_. The equation estimated by Shkolnikov et al. (2003) for obtaining *A`*_85+_ is given below:

3 $$ A{`}_{85+}=\left(1/\left({l}_{85}\right)2\operatorname{}\right)\kern0.5em {\displaystyle {\sum}_{x=85}^{109}\kern0.5em \left[{\left({l}_{x+1}\right)}^2\kern0.5em +\kern0.5em  A{`}_{\mathrm{x}}\kern0.5em \left({\left({l}_x\right)}^2-\kern0.5em {\left({l}_{x+1}\right)}^2\right)\right]} $$

Substituting the values *l*_*x*_, *A*_*x*_, and *A`*_*x*_ in formula (3) Shkolnikov et al. (2003) obtained the following equations for *A`*_85+_.

4 $$ \begin{array}{l} A{`}_{85+}=-0.440+0.680*{\mathrm{e}}_{85}\left(\mathrm{for}\ \mathrm{women}\right),\\ {} A{`}_{85+}=-0.227+0.626*{\mathrm{e}}_{85}\left(\mathrm{for}\ \mathrm{men}\right)\end{array} $$

The SRS until the year 1994 used to provide age-specific death rates for broad 5-year age group, the last age being 70+. From the calendar year 1995, the SRS has extended the last age group to 85+. Hence, to carry out a trend analysis, we made the last age group as 70+ for all the years included in the study. Then we used the single-year mortality data starting from age 70 to 110 years obtained from the 334 life tables from the Berkeley Mortality Database to estimate *A`*_70+_. We used formula (3) to estimate *A`*_70+_. The equations for *A`*_70+_ are given below:

5 $$ \begin{array}{l} A{`}_{70} = -2.104+0.860*{\mathrm{e}}_{70}\left(\mathrm{for}\ \mathrm{women}\right),\\ {} A{`}_{70}=-1.336+0.780*{\mathrm{e}}_{70}\left(\mathrm{for}\ \mathrm{men}\right)\end{array} $$

Finally, we used Eqs. (4) and (5) on SRS datasets for the years 1995 and later to examine whether the two equations yield substantially different results. The Gini coefficients obtained from the two equations were close. Since the differences between the two coefficients were minimal, we used formula (5) to estimate *A`*_70_.

### Decomposition of Gini coefficient by age groups

Shkolnikov et al. (2003) have developed a new formula for the decomposition of differences between Gini coefficients by age and causes of death. The general procedure for decomposition by age of a difference in two Gini coefficients *G*_0_ and *G*`_0_ is

$$ \delta ={G}_0- G{`}_0={G}_0{M}^{\Big[ x}{{}_{i+1}}^{\Big]}{\textstyle \hbox{-} }{G}_0{M}^{\Big[ x}{{}_i}^{\Big]} $$

where *M* ^[*xi*]^ is a vector of age-specific mortality rates with elements *m*`_*x*_ for *x* < = *x*_*i*_ and *m*_*x*_ for *x > =x*_*i*_. It determines a stepwise replacement of one mortality schedule by another one, beginning from the youngest to the oldest age group.

### Decomposition of Gini coefficient by causes of death

Let *δ*_(*xi,xi+n*)_ is the absolute change in *G*_0_ due to age group (*x*_*i*_, *x*_*i+n*_), *m*_*xi|xi+n*, *j*_ and *m*`_*xi|xi+n,j*_ are the *j*th cause-specific death rates for age group (*x*_*i*_, *x*_*i+n*_) then age- and cause-specific change in Gini coefficient is given by(Shkolnikov et al. 2003).

$$ {\delta}_{\left( xi,\  xi + n\right)\ \Big| j} = \frac{m_{\left( xi, xi+ n\right)\left| j-\right.} m{`}_{\left( xi, xi+ n\right)\left| j\right.}}{m_{\left( xi, xi+ n\right)- m{`}_{\left( xi, xi+ n\right)}}}\kern1em *\kern1em {\delta}_{\left( xi, xi\kern0.5em +\kern0.5em  n\right)} $$

## Results

Tables 1 and 2 show life expectancy at birth and Gini coefficient (*G*_0_) of life expectancy at birth in India and 15 major states. The life expectancy at birth has increased substantially in India in the past three decades. For men, it has risen from 51 years in 1981 to 66 years in 2011, and for women, it has increased from 55 years in 1981 to 71 years in 2011. On the contrary, *G*_0_ of life expectancy registered a decline during this period. For men, *G*_0_ declined from 32 in 1981 to 19 in 2011. Similarly, for women, the *G*_0_ shows a reduction of 9 points. The state-level analysis suggests that the *G*_0_ for men in 1981 is highest in Uttar Pradesh (32) and lowest in Kerala (18). Interestingly, the state-level variations in *G*_0_ narrowed down over the decades. In 2011, the *G*_0_ for Karnataka, Maharashtra, and Kerala is substantially lower than the national average. In comparison, the *G*_0_ for Haryana, Rajasthan Uttar Pradesh, Madhya Pradesh, and Odisha is substantially higher than the national average. The picture is similar for women. Notably the *G*_0_s were higher for women than for men.

**Table 1:** Life expectancy (LE) at birth and Gini coefficient of LE from 1981 to 2011, India and major states, men

	LE				*G*_0_*100			
	1981	1991	2001	2011	1981	1991	2001	2011
North								
Punjab	62.52	63.75	68.41	69.62	23.21	21.39	21.92	20.16
Haryana	59.66	62.36	65.57	67.34	28.10	22.62	23.77	21.04
Rajasthan	53.05	60.55	67.16	71.67	28.09	28.12	28.39	25.40
Central								
Uttar Pradesh	50.81	57.28	63.49	65.65	32.43	26.32	27.79	23.83
Madhya Pradesh	49.43	54.58	58.76	62.66	31.90	27.35	24.09	20.87
North East								
Assam	52.93	55.41	57.76	62.23	27.17	24.58	22.75	19.92
East								
West Bengal	55.21	61.01	64.76	68.73	23.73	21.63	19.60	17.11
Odisha	52.24	54.77	59.20	63.78	30.42	27.69	24.13	20.71
Bihar	56.50	60.05	64.88	68.87	25.75	24.10	22.62	20.17
West								
Gujarat	53.93	59.95	63.81	66.32	28.10	21.94	21.26	19.29
Maharashtra	59.24	63.38	65.19	69.55	22.72	20.13	19.48	16.91
South								
Andhra Pradesh	55.41	59.38	62.38	65.37	24.65	21.74	21.05	18.61
Karnataka	59.36	60.47	63.02	66.67	23.21	22.06	21.46	18.13
Kerala	64.52	68.04	70.23	71.61	18.40	15.47	14.80	14.79
Tamil Nadu	55.83	61.06	65.47	71.98	26.05	20.12	18.99	16.46
India	50.76	59.31	62.86	66.29	32.47	23.48	22.09	19.30

**Table 2:** Life expectancy (LE) at birth and Gini coefficient of LE from 1981 to 2011, India and major states, women

	LE				*G*_0_*100			
	1981	1991	2001	2011	1981	1991	2001	2011
North								
Punjab	63.53	67.39	67.65	75.18	26.92	23.59	23.43	21.37
Haryana	57.31	63.63	69.46	72.22	29.65	25.00	27.75	23.20
Rajasthan	54.69	58.85	63.20	65.62	32.22	23.74	23.47	20.23
Central								
Uttar Pradesh	48.38	55.65	61.06	66.93	38.75	30.59	28.71	24.18
Madhya Pradesh	49.33	54.16	59.87	66.40	34.54	30.06	27.60	22.92
North East								
Assam	52.43	55.46	60.04	65.55	29.49	25.83	25.71	22.25
East								
West Bengal	55.71	62.12	68.10	72.96	26.95	22.95	21.08	19.88
Odisha	51.30	53.90	61.64	67.45	31.06	29.85	25.40	22.43
Bihar	48.89	58.87	65.95	68.16	33.11	27.73	25.24	19.54
West								
Gujarat	57.37	63.43	69.05	72.65	29.70	24.62	25.13	23.04
Maharashtra	60.89	65.17	69.17	74.92	25.72	22.40	20.99	18.92
South								
Andhra Pradesh	58.62	62.34	67.64	71.80	25.93	22.27	22.24	21.48
Karnataka	60.70	64.70	69.92	72.20	26.30	23.92	23.21	19.73
Kerala	70.19	74.73	77.01	79.33	19.05	16.46	17.27	18.48
Tamil Nadu	56.18	64.00	69.13	68.83	27.25	20.95	20.15	17.27
India	54.65	60.24	65.59	70.84	30.97	26.16	24.63	21.84

Tables 3 and 4 present the age-specific contributions to the decrease in *G*_0_ between 1981 and 2011 in India and 15 major states for men and women. At the all India level, the mortality decline in the age group 0–1 year contributed to 53% of the decline in *G*_0_ for men. Furthermore, the mortality decline in the age group 1–4 reduced the *G*_0_ by 52%. For women, the mortality decline in the age group 0–1 year contributed to 55% of the decline in *G*_0_. The mortality decline in the age group 1–4 further reduced the *G*_0_ by 49%. Interestingly, the mortality decline in the age group 60+ tended to increase the *G*_0_ during this period. Mortality decline in 60+ age group contributed to 16% and 33% increase in *G*_0_ for men and women during 1981–2011 respectively.

**Table 3:** Age-specific contributions to the decrease in Gini coefficient between 1981 and 2011, India and major states, men

	% change in *G*_0_				
	0–1	1–4	5–14	15–59	60+
North					
Punjab	145.38	54.31	15.88	−7.34	−108.23
Haryana	67.66	42.80	12.42	−0.87	−22.02
Rajasthan	105.45	136.89	44.11	38.92	−225.38
Central					
Uttar Pradesh	72.44	45.78	11.13	5.51	−34.86
Madhya Pradesh	59.10	43.38	9.51	0.38	−12.37
North East					
Assam	56.13	33.30	11.32	7.71	−8.45
East					
West Bengal	90.53	56.95	8.09	15.90	−71.47
Odisha	78.95	28.78	7.02	8.17	−22.92
Bihar	75.01	31.26	17.00	20.18	−43.45
West					
Gujarat	71.95	41.61	11.80	7.96	−33.33
Maharashtra	90.00	53.10	16.81	4.96	−64.87
South					
Andhra Pradesh	77.23	56.62	16.99	5.14	−55.97
Karnataka	67.71	54.31	13.12	−1.01	−34.14
Kerala	78.75	46.36	15.29	19.26	−59.65
Tamil Nadu	72.34	46.09	15.45	21.78	−55.65
India	53.24	52.26	6.69	3.83	−16.03

**Table 4:** Age-specific contributions to the decrease in Gini coefficient between 1981 and 2011, India and major states, women

	% change in *G*_0_				
	0–1	1–4	5–14	15–59	60+
North					
Punjab	76.77	51.93	10.86	16.27	−55.84
Haryana	68.07	62.75	10.27	20.50	−61.59
Rajasthan	29.58	48.09	10.68	9.79	1.86
Central					
Uttar Pradesh	48.83	45.11	8.63	11.01	−13.58
Madhya Pradesh	45.28	51.23	12.35	13.03	−21.89
North East					
Assam	48.48	38.65	15.09	26.30	−28.51
East					
West Bengal	60.27	56.60	13.50	34.10	−64.46
Odisha	71.01	36.48	12.64	18.13	−38.27
Bihar	48.82	43.72	10.80	16.88	−20.23
West					
Gujarat	77.94	37.34	15.28	22.23	−52.78
Maharashtra	59.28	49.69	11.01	21.55	−41.53
South					
Andhra Pradesh	63.04	75.40	22.58	29.35	−90.36
Karnataka	38.08	53.68	16.66	19.84	−28.27
Kerala	270.39	250.41	68.64	149.24	−638.69
Tamil Nadu	53.80	45.75	10.10	10.62	−20.27
India	55.35	49.04	11.94	17.15	−33.48

The state-wide variations in the age-specific contributions to decrease in *G*_0_ are stark. The contribution of the mortality decline in the age group 1–4 for men ranged between as high as 137% in Rajasthan and as low as 29% in Odisha. For women, this contribution ranged between 250% in Kerala and 36% in Odisha. Likewise, the contribution of the age group 0–1, for both men and women, also varied considerably across the states. For men, it ranged between as low as 56% in Assam and as high as 145% in Punjab. For women, the contribution of 0–1 year varied between as low as 30% in Rajasthan and as high as 270% in Kerala. Like India, the decline in mortality in the 60+ age group tended to increase the *G*_0_ for both men and women in the 15 major states. However, the magnitude of increase is significantly different in men and women in various states. For example, in Kerala women, the decline in mortality in 60+ contributed to 639% increase in *G*_0_ during 1981–2011. In comparison, the contribution of decline in mortality in 60+ to increase in *G*_0_ for Kerala men is only 60%.

**Fig. 1:**
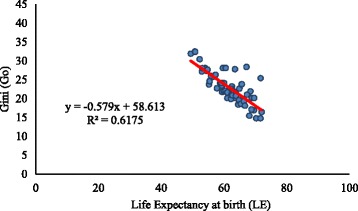
Relationship between life expectancy at birth and Gini coefficient, men, India

**Fig. 2:**
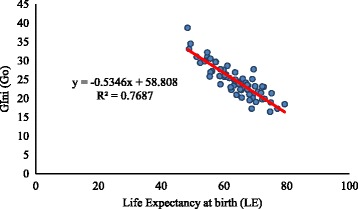
Relationship between life expectancy at birth and Gini coefficient, India, women

Figures 1 and 2 indicate the pattern of association between *G*_0_ and the life expectancy at birth for men and women respectively. The results show a negative relationship between life expectancy at birth and *G*_0_, thus indicating that an increase in life expectancy at birth is accompanied by a decrease in inequality in length of life. The relationship is similar for men and women. The correlation coefficient between life expectancy at birth and Gini coefficient by states is −0.79 for men and −0.88 for women. Same life expectancies for many states correspond to different levels of Gini coefficients. For example, for men, the life expectancy at birth in Kerala and Rajasthan were 71. 6 years in 2011. However, the Gini coefficients are 14.8 and 25.4 respectively. Likewise, for women, the life expectancy at birth in Karnataka and Haryana was 72.2 years in 2011. But the Gini coefficients for these two states are 19.7 and 23.2 respectively. The Gini coefficients for the men population are substantially higher than those predicted by life expectancy at birth in Rajasthan in years 2001 and 2011 and in Uttar Pradesh in the year 2001. For women population, the Gini coefficient is substantially higher than that predicted by life expectancy at birth in Haryana in the year 2001. In comparison, the Gini coefficient was lower than the predicted value in Tamil Nadu in 2011.

**Fig. 3:**
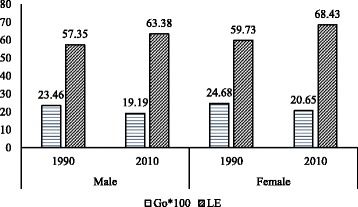
Life expectancy at birth and Gini coefficient for men and women, India, 1990 and 2010

Data from the Global Burden of Disease study (GBD) also shows a decline in *G*_0_ values for both men and women during 1990–2010. For men, the *G*_0_ declined from 23 to 19. Similarly, for women, it declined from 25 in 1990 to 21 in 2010. While women have the upper hand in life expectancy at birth, the *G*_0_ is higher for women than men in both the rounds. Moreover, the male-female gap in *G*_0_ has widened between 1990 and 2010 indicating increasing sex differential in inequality in life expectancy at birth (Fig. 3).

Table 5 shows the age-specific contributions to the difference in *G*_0_ for men and women in India in 1990 and 2010. In 1990, the male-female difference in mortality in the age 60+ contributed to 107% of the male-female gap in *G*_0_. In 2010, this contribution increased to 164%. The male-female gap in mortality in the age group 0–1 decreased the male-female gap in *G*_0_ in both 1990 and 2010. However, the contribution of male-female mortality gap in the age group 0–1 year declined between 1990 and 2010. In 1990, the contribution of 1–4 age group was also substantial. However, in 2010, the contribution of age group 1–4 reduced to only 8%. Notably, in 1990, the male-female difference in mortality in the age group 15–59 years tended to increase the male-female gap in *G*_0_. In contrast, in 2010, male-female mortality gap in the age group 15–59 years tended to reduce the male-female gap in *G*_0_.

**Table 5:** Age-specific contributions to the difference in Gini coefficient between men and women, India, 1990 and 2010

	% change in *G*_0_	
Age groups	1990	2010
0–1	−64.29	−25.63
1–4	24.91	7.68
5–14	11.32	−0.09
15–59	20.92	−46.37
60+	107.14	164.40

**Fig. 4:**
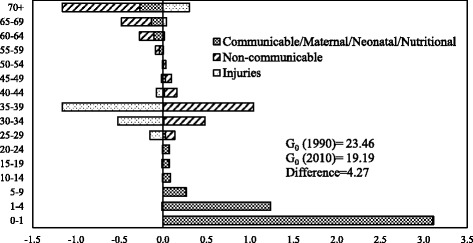
Decomposition of the differences in Gini coefficient between 1990 and 2010 by age and cause of death, men, India

**Fig. 5:**
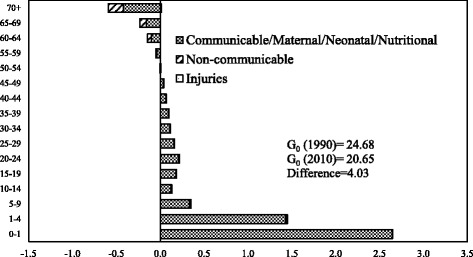
Decomposition of the differences in Gini coefficient between 1990 and 2010 by age and cause of death, women, India

Figures 4 and 5 show age- and cause-specific contributions to the decrease in *G*_0_between 1990 and 2010 for men and women respectively. The inequality in length of life for both men and women declined between 1990 and 2010. The decline in mortality in the age group 0–1 year contributed the maximum to the decrease in *G*_0_ between 1990 and 2010. The contribution of age group 0–1 year to the decline in *G*_0_ was larger for men as compared to women. Older ages contributed negatively to the decrease in *G*_0_ for both men and women. The contribution of older ages was more among the men than in women. The communicable, maternal, neonatal, and nutritional diseases during infancy and early childhood contributed significantly to the decline in *G*_0_ for both men and women. The contribution of this major category was more in men than in women in the age group 0–1. In age group 1–4, the contribution of communicable, maternal, neonatal, and nutritional diseases to the decline in *G*_0_ was more in women than in men. In comparison, the noncommunicable diseases and communicable, maternal, neonatal, and nutritional diseases at older ages contributed negatively to the decline in *G*_0_ for both men and women. The contribution was more among men than in women. Notably, for men, injuries in the age group 30–39 contributed negatively to the decline in *G*_0_ between 1990 and 2010.

**Fig. 6:**
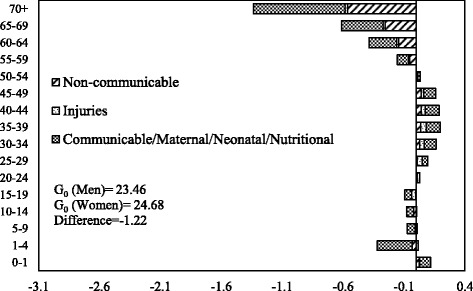
Decomposition of the differences in Gini coefficient between men and women by age and cause of death, India, 1990

**Fig. 7:**
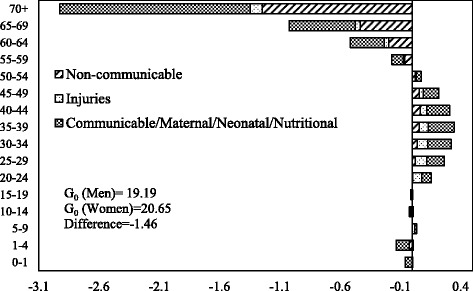
Decomposition of the differences in Gini coefficient between men and women by age and cause of death, India, 2010

The decomposition of male-female difference in *G*_0_ by age and causes of death in 1990 and 2010 is shown in Figs. 6 and 7 respectively. The communicable, maternal, neonatal, and nutritional diseases during early childhood and older ages and the noncommunicable diseases at ages 55 years or above are the major contributors to the male-female difference in *G*_0_ in 1990. Also, communicable, maternal, neonatal, and nutritional diseases in the age group 30–49 contributed significantly to the male-female difference in *G*_0_. In 2010, the pattern of the contribution of causes of deaths was similar to that of 1990. However, the magnitude of contribution has changed significantly. The communicable, maternal, neonatal and nutritional, and noncommunicable diseases in older ages are the major contributors to the male-female difference in *G*_0_. The contribution of communicable, maternal, neonatal, and nutritional diseases at infancy and early childhood to the male-female difference in *G*_0_ is lower in 2010 compared to the contribution in 1990. In comparison, the contribution of noncommunicable diseases at ages 55 years or more to the male-female difference in *G*_0_ is higher in 2010 than in 1990. Notably, injuries at ages from 20 to 39 years also contributed to the male-female difference in *G*_0_ in 2010.

## Discussion

The above analysis shows the trends in Gini coefficient (*G*_0_) in Indian men and women during 1981 and 2011. The study also presents the decomposition of the difference in Gini in Indian men and women during 1990 and 2010 by age and causes of death. The total decrease in *G*_0_ *100 over the period 1981 to 2011 is 13 points among the men and 9 points among the women indicating that the inequality has decreased more in men than in women during this period. The GBD-based results show that decrease in *G*_0_*100 is almost equal for men and women during 1990 to 2010. The proportion of decrease in *G*_0_ due to decline in mortality in the age group 0–1 year is highest for both men and women indicating that rapid improvements in mortality during infancy has led to equalization of ages at death. The decline in mortality rates during infancy contributed to 53 and 55% of the decline in *G*_0_ for men and women respectively. The scenario is opposite in older age groups where mortality decline in men and women aged 70 years or more has increased the level of *G*_0_ in 2011 in comparison to 1981. While female elderly contributed to 33% in the increase in *G*_0,_ the male elderly contributed only 16%. The mortality decline at ages 15–59 years helped more in equalizing the age at death in women compared to men.

A comparison of our results with that of other existing studies suggests that our estimated Gini for India is close to that in neighboring countries like Bangladesh. The estimated value of Gini for women in Matlab (Bangladesh) in 1995 was 0.22 (Shkolnikov et al. 2003). Our estimates for the same period are around 0.25. Notably, the life expectancy at birth in 1995 in Matlab (Bangladesh) and India were 63.5 and 63.0 years respectively. A comparison of our estimated Gini with the Gini for select European countries suggests that the Gini for Indian women for 1995 is considerably higher than that in European countries like Russia and Sweden (Shkolnikov et al. 2003). The Gini for Russian and Swedish women were 0.13 and 0.08 respectively. Findings also suggest that the Gini for women in Kerala (0.16) in 1995 is close to that in Russian women. It is worthwhile to note that the life expectancy at birth in 1995 in Russian and Swedish women was 72 and 82 years (Shkolnikov et al. 2003). During this period, the life expectancy at birth in Kerala women was 76 years. The comparisons presented above and the negative relationship between life expectancy at birth and Gini reveal that our estimated Gini in India and 15 major states are plausible.

Studies have established that even if the levels of life expectancy are similar, different populations can have varying levels of inequality in length of life (Smits and Monden 2009). This analysis reveals that although life expectancy has shown drastic improvement in past decades in India, not much has changed in inequality in length of life. The improvement in life expectancy is much higher than the improvement in *G*_0_. Like previous studies, our analysis also reveals that rapid reduction in infant and child mortality has caused great equalization of ages at death (Shkolnikov et al. 2003). Due to improvement in health care services and constant effort by the Indian Government, under-5 mortality in India has shown a considerable decline. Among men, under-5 mortality has declined from 297 deaths per 1000 live births in 1981 to 53 deaths per 1000 live births in 2011. Similarly, among women, it has decreased from 180 deaths per 1000 live births in 1981 to 61 deaths per 1000 live births in 2011 (RGI 1985, 2013). In contrast, the mortality at ages 70 years or more has led to an increase in inequality in ages at death. Mortality in the age group 70 or more has declined from 93 to 66 among women and from 103 to 82 among men during 1981–2011. However, this decrease has not been monotonic (RGI 1985, 2013).

A key finding of our study is the marked differentials in estimated Gini for men and women in the major states of India. Indeed, the estimated Gini for both men and women in the recent period is higher in the north and central Indian states compared to the south Indian states. For example, the estimated Gini for men in 2011 in Punjab, Haryana, and Rajasthan are 0.20, 0.21, and 0.25 respectively. This compares with 0.18, 0.15, and 0.16 in Karnataka, Kerala, and Tamil Nadu respectively. The estimated Gini in Uttar Pradesh and Madhya Pradesh are 0.24 and 0.21 respectively. The estimated Gini in Odisha is also high (0.21). It is important to note that the infant and child mortality in the northern, central, and eastern India is markedly higher than that in southern India. The infant mortality rates in Rajasthan, Uttar Pradesh, Madhya Pradesh, and Odisha in 2013 were 47, 50, 54, and 51 respectively (RGI 2014). In comparison, the infant mortality rates in Karnataka, Kerala, and Tamil Nadu were 31, 12, and 21 respectively.

Several studies in the past have indicated that the burden of noncommunicable diseases is increasing in India and is emerging as the most important cause of death (Sharma 2013; Upadhyay 2012). Recent data also suggests that age-specific death rates (ASDRs) from communicable diseases have decreased from 551 deaths per 100,000 population in 1990 to 284 deaths per 100,000 population in 2010. On the other hand, ASDRs due to noncommunicable diseases have increased from 455 deaths per 100,000 population in 1990 to 470 deaths per 100,000 population in 2010 among men. Among women, the rate was 390 deaths per 100,000 population in 1990 and 395 deaths per 100,000 population in 2010. The same pattern is evident in injuries too. Injury-specific ASDRs have increased from 84 deaths per 100,000 population in 1990 to 93 deaths in 2010 (GBD 2013). Estimates also suggest that noncommunicable diseases account for 60% of the total deaths in India (World Health Organization 2014). Our study also shows that the contribution of noncommunicable diseases to the male-female gap in *G*_0_ has increased between 1990 and 2010. Furthermore, the noncommunicable diseases at older ages have contributed to increasing the inequality in length of life in India for both men and women.

Limitations of our study must also be noted. First, the last age group included in our analysis is 70+. Shkolnikov et al. (2003) have shown that the estimation of *G*_0_ using aggregated mortality rates at older ages rather than real mortality rates might get affected due to the increase in the proportion of deaths at older ages. We used 70+ as the last age group in our analysis because of the non-availability of disaggregated data during the period 1981 to 1994. Using data that ends at 70+ might cause errors in the computation of *G*_0._ To quantify the magnitude of error that might have occurred due to the use of 70+, we estimated *G*_0_ using 85+ for the period 1995 to 2011 for which data up to 85+ is available. Then we compared the *G*_0_ obtained by using 70+ and 85+ for the period 1995 to 2011. The comparison suggested only minor differences in the *G*_0_ estimated using 70+ and 85+. Shkolnikov et al. (2003) have also shown that currently the error is small and hence can be ignored. But in future, if mortality rates at older ages continue to increase it will be necessary to use real mortality data for estimation of *G*_0_ (Shkolnikov et al. 2003). Second, we could not do a trend analysis for larger time periods due to non-availability of quality Indian life tables before 1980. Third, the age- and cause-specific decomposition of *G*_0_ is restricted only to 1990 and 2010. This is due to the non-availability of data on causes of deaths by age in India for other years. Fourth, we could not do the age- and cause-specific decomposition of *G*_0_ for the states of India due to non-availability of data on causes of death by age for the 15 major states of India in the GBD 1990 and 2010. An important limitation of the study is that the analysis restricts to only three major categories of causes of death (i.e., communicable, maternal, neonatal, and nutritional; injuries; noncommunicable). We included only three major categories because the aim of this study is to broadly identify the sources of inequality in length of life in India. The three major categories provided us robust information about the contribution of these causes of deaths to changes in *G*_0_ over time.

The paper has strengths that outweigh the limitations. This is by far the first study which focuses solely on inequality in length of life in India and selected states. Until now little was known about the nature and pattern of inequality in life expectancy in India and its major states. The age-wise decomposition of Gini indicates that the largest contributors in decreasing inequality in life expectancy are the early childhood ages. On the other hand, mortality at older ages has negatively influenced inequality in life expectancy. This may be due to the excessive importance given to reduce child mortality. The study points out that there is a need for a shift in the focus of mortality studies in India. Several studies in the past have revealed that noncommunicable diseases account for a substantial proportion of disease burden in India (Sharma 2013; Upadhyay 2012; World Health Organization 2014). Our study shows that not only the deaths from noncommunicable diseases are rising in numbers, but also noncommunicable diseases at older ages are also responsible for inequality in life expectancy.

## Conclusion

Overall it can be said that while gains in life expectancy have been remarkably steady both overall and across states of India, gains against life span variance have been scarcer. Our findings imply that the absolute level of length of life is not an informative measure on its own. Hence, apart from studying improvements in mortality levels, future studies must analyze the inequality in mortality to assess the performance of societies on length of life.
